# *Terminalia chebula* Retz. Fruit Extract Promotes Murine Hair Growth by Suppressing 5α-Reductase and Accelerating the Degradation of Dihydrotestosterone

**DOI:** 10.3390/biomedicines13112584

**Published:** 2025-10-22

**Authors:** Ting Cui, Xiaoqing Wang, Qi Wu, Ye Zhong, Fenglou Wang, Yue Zou, Yushu Wang, Shanshan Jiang, Gang Ma

**Affiliations:** 1 Key Laboratory for the Genetics of Developmental and Neuropsychiatric Disorders (Ministry of Education), Bio-X Institutes, Shanghai Jiao Tong University, Shanghai 200240, China; 2 R&D Center, Shanghai CHANDO Group Co., Ltd., Shanghai 200233, China

**Keywords:** AGA, *Terminalia chebula* fruit extract, AKR1C, dihydrotestosterone, 5α-reductase

## Abstract

**Background/Objectives:** Androgenetic alopecia (AGA) is the most common hair loss disorder in dermatological practice. Its primary pathogenesis involves the conversion of testosterone to dihydrotestosterone (DHT) by type II 5α-reductase upon reaching dermal papilla cells (DPCs). DHT impairs DPCs’ activity and inhibits hair growth. Although the FDA-approved drugs finasteride and minoxidil show certain efficacy, they are also associated with severe side effects. This study aims to explore the effects of *Terminalia chebula* fruit extract (TCFE) on hair growth and its underlying molecular mechanisms. **Methods**: We investigated the therapeutic potential of TCFE in hair follicle regeneration, employing a multi-level experimental approach combining in vitro analyses of DPCs, in vivo animal models of AGA, and ex vivo cultures of human hair follicles and scalp tissue. **Results**: First, RNA-seq analysis and RT-PCR validation revealed that TCFE treatment activated the Wnt and TGF-β3 signaling pathways in DPCs, particularly upregulating the AKR1C gene family, which is involved in DHT metabolism. TCFE also potently inhibited type II 5α-reductase activity and mitigated DHT-induced damage to DPCs. In an AGA mouse model, TCFE reversed the AGA phenotype with efficacy comparable to finasteride. However, unlike finasteride, TCFE specifically enhanced the expression of AKR1C1 and AKR1C3, indicating a distinct mechanism. Finally, in ex vivo organ cultures, TCFE suppressed hair follicle cell apoptosis, promoted proliferation, and thereby stimulated hair growth. **Conclusions**: These findings suggest that TCFE is a promising natural treatment for AGA, likely acting through multiple mechanisms, including Wnt pathway activation, 5α-reductase inhibition, and enhanced DHT degradation.

## 1. Introduction

In modern society, the accelerating pace of life and increasingly intense academic and career pressures have jointly contributed to a growing prevalence of hair loss—a trend particularly pronounced among younger populations. Androgenic alopecia (AGA), the most prevalent form of pattern hair loss, is pathophysiologically characterized by progressive hair follicle miniaturization due to a shortened anagen phase and prolonged telogen phase in the hair cycle [1]. This process manifests clinically as reduced hair density, accompanied by seborrheic symptoms including scalp scaling and excessive sebum production. Epidemiologic studies reveal significant gender and ethnic variations: while Caucasian populations exhibit the highest prevalence (approximately 80% in males and 40–50% in females), Asian and African cohorts demonstrate comparatively lower incidence rates [2,3]. These disparities likely reflect complex interactions between genetic predisposition, androgen sensitivity, and environmental or lifestyle factors.

AGA is linked to elevated levels of dihydrotestosterone (DHT), produced via the enzymatic conversion of testosterone under the catalysis of 5α-reductase. Upon binding to the androgen receptor (AR), DHT forms a hormone-receptor complex that activates target gene expression [4]. This process results in a shortened anagen phase, accelerated apoptosis of hair follicle cells, follicular miniaturization, and ultimately leads to hair loss [5,6]. Although testosterone also demonstrates affinity for AR, its binding affinity is considerably weaker compared to DHT [4]. Studies have shown that both 5α-reductase and androgen receptor expression levels are upregulated in individuals with AGA [7].

Current therapeutic approaches for AGA primarily involve pharmacological interventions and surgical procedures. Two drugs approved by the U.S. Food and Drug Administration (FDA) for AGA treatment are minoxidil and finasteride [8]. Minoxidil is a topical vasodilator that enhances follicular blood perfusion to promote hair growth [9]. Finasteride is an oral 5α-reductase inhibitor that reduces DHT synthesis to mitigate hair loss progression [10]. For advanced cases, follicular unit transplantation (FUT) has emerged as the gold-standard surgical intervention, involving the relocation of androgen-resistant hair follicles to affected scalp regions [11]. While pharmacological therapy demonstrates optimal efficacy during early AGA stages, combination approaches incorporating both medical and surgical modalities are typically required for advanced cases. However, these conventional treatments present significant limitations: pharmacotherapy necessitates indefinite maintenance with potential adverse effects, including finasteride-associated sexual dysfunction and psychological sequelae, while surgical transplantation offers only temporary cosmetic improvement without altering disease progression [12,13]. Furthermore, surgical candidacy is often restricted by insufficient donor hair availability in severe alopecia cases. These limitations have spurred investigation into novel therapeutic strategies, particularly in regenerative medicine and natural product research. Emerging regenerative approaches include adipose-derived stem cell therapy [14] and platelet-rich plasma (PRP) treatments [15]. Additionally, natural products like saw palmetto, grape seed, melatonin, marine extracts, rosemary oil, pumpkin seed oil, and cannabidiol oil are being investigated for their potential in promoting hair growth for AGA treatment [16,17].

*Terminalia chebula* Retz., a widely used medicinal plant from the Combretaceae family in traditional medicine [18], is particularly valued for its fruit, which contains a diverse array of bioactive compounds. Extensive pharmacological studies have demonstrated its significant antioxidant, anti-inflammatory, antibacterial, anti-aging, and antiviral properties [19,20]. The fruit extract is especially rich in hydrolysable tannins, including chebulagic acid and chebulinic acid, which have been shown to possess potent anti-inflammatory [21,22] and antioxidant activities [23,24,25]. The ethanol extract of *T. chebula* fruit can protect the skin from UVB-induced damage by downregulating matrix metalloproteinases (MMP-1 and MMP-13) while upregulating type I procollagen synthesis, thereby preserving skin structural integrity [26]. Through advanced analytical methods, it has been revealed that *T. chebula* fruit extract promotes angiogenesis in diabetic wounds via the PI3K/AKT and HIF-1α signaling pathways, thereby enhancing wound healing [27]. These findings highlight the multifaceted therapeutic potential of *T. chebula* in dermatological applications, including maintenance of cutaneous homeostasis, anti-aging interventions, and photodamage reversal. While its benefits for skin health are well-established, the effects of *T. chebula* on hair follicle biology and hair growth remain largely unexplored.

In this study, we investigated the therapeutic potential of *T. chebula* fruit extract (hereafter referred to as TCFE) in hair follicle regeneration, employing a multi-level experimental approach combining in vitro analyses of DPCs, in vivo animal models of AGA, and ex vivo human hair follicle organ culture. Our integrated investigation reveals that TCFE promotes hair growth via activating Wnt/β-catenin signaling and TGF-β signaling, and attenuates the early-stage AGA pathophysiology through inhibiting 5α-reductase activity and increasing DHT metabolism.

## 2. Materials and Methods

### 2.1. Materials

Testosterone (HPLC purity > 98%), finasteride (HPLC purity > 98%), and testosterone propionate (HPLC purity > 98%) were purchased from Aladdin Biochemical Technology Co., Ltd. (Shanghai, China). Insulin, hydrocortisone, and L-glutamine were acquired from Sigma-Aldrich (St. Louis, MO, USA). TCFE and DPCs were obtained from JALA Corporation (Shanghai, China). CCK-8 kit was purchased from Beyotime Biotechnology (Shanghai, China).

### 2.2. TCFE Preparation and LC-MS Analysis

The *Terminalia chebula* Fruit Extract was prepared according to the following procedure: Dried fruits of *Terminalia chebula* were first pulverized into a fine powder. The resulting powder was then subjected to aqueous extraction with intermittent stirring (2–5 h). The extraction mixture was subsequently filtered to collect the filtrate. Finally, the filtrate was dried to obtain the final *Terminalia chebula* fruit extract. The LC-MS (Liquid Chromatograph–Mass Spectrometer, Thermo-Orbitrap-QE) analysis, including the compositional testing and characterization of TCFE, was performed by Shanghai OE Biotech Co., Ltd. (Shanghai, China). Mass spectrometric data acquisition was performed in both positive and negative ion scanning modes (POS/NEG).

### 2.3. Cell Culture and RNA Extraction

DPCs were cultivated in Dulbecco’s modified Eagle’s medium (Gibco, Grand Island, NY, USA, 11965092) with 10% FBS, 1% L-glutamine, and 1% penicillin/streptomycin solution in a humidified incubator supplemented with 95% air/5% CO_2_ at 37 °C. The cells were passaged every 3 days to maintain optimal growth conditions. The culture medium was replaced every 3 days. For all experimental treatments, DPCs were cultured for a standardized duration of 72 h prior to RNA extraction. Total RNA was then isolated using the TRIzol reagent according to the manufacturer’s protocol, with subsequent quality verification by spectrophotometry (A260/A280 ratio > 1.8) and agarose gel electrophoresis to ensure RNA integrity.

### 2.4. CCK-8 Assay

For cell viability assessment, cells were plated in 96-well plates at an initial density of 2 × 10^3^ cells/well in 100 μL complete medium. Following a 24 h attachment period, cells were exposed to varying concentrations of TCFE (0.0005–0.1%) for 24 h under standard culture conditions (37 °C, 5% CO_2_). After treatment, the medium was carefully aspirated, and cells were gently washed twice with phosphate-buffered saline (PBS, pH 7.4) to remove residual compounds. Subsequently, 100 μL of fresh medium containing 10% (*v*/*v*) CCK-8 reagent was added to each well, followed by incubation for 2 h at 37 °C. The optical density was measured at 450 nm using a multimode microplate reader (BioTek Instruments, Winooski, VT, USA). The calculation formula for cell viability is Cell viability = (*A*1 − *A*0)/(*A*2 − *A*0) × 100%, where *A*0 represents the absorbance of medium group; *A*1 represents the absorbance of cell sample group; *A*2 represents the absorbance of the blank control group.

### 2.5. Animal Experiments

Male C57BL/6 mice (7–8 weeks, 18–22 g) were maintained in specific pathogen-free (SPF) facilities under controlled environmental conditions (22 ± 1 °C, 50 ± 10% humidity, 12 h light/dark cycle) with ad libitum access to standard chow and autoclaved water. All experimental procedures were conducted in compliance with the ARRIVE guidelines and approved by the Institutional Animal Care and Use Committee (IACUC) of Shanghai Jiao Tong University, with strict adherence to the 3Rs principles (Replacement, Reduction, Refinement). Before the experiment, we performed depilation on these mice and divided the mice into 4 groups (*n* = 3 per group): blank group (untreated), AGA-model group (Mice receiving daily subcutaneous injections of testosterone propionate), Finasteride group (Testosterone-treated mice receiving concurrent finasteride (50 mg/kg/day) via oral gavage), TCFE 0.5% group (Testosterone-treated mice with topical application of 0.5% TCFE suspension). Testosterone propionate was dissolved in sterile corn oil to prepare a 10 mg/mL solution, which was sonicated for 15 min at 37 °C to ensure complete dissolution. Mice were anesthetized using an isoflurane vaporizer system (2% isoflurane in oxygen) prior to subcutaneous administration. Testosterone propionate (5 mg/kg) was administered subcutaneously daily, with injection sites rotated systematically to prevent local corn oil accumulation. Vehicle control mice received equivalent volumes of sterile corn oil alone. Throughout the 14-day treatment period, skin condition and hair growth were monitored daily.

### 2.6. Histology and Immunofluorescence

Dorsal skin samples from all experimental groups of AGA model mice and human scalp tissues containing hair follicles were fixed in 4% formaldehyde solution for 24 h at 4 °C, followed by standard paraffin embedding. Longitudinal sections (6 μm thickness) were prepared using a rotary microtome. After deparaffinization with xylene and graded ethanol hydration, antigens were retrieved with sodium citrate buffer (pH 6.0) at 98 °C for 20 min. The sections were processed for Hematoxylin and eosin (H&E) staining, immunofluorescence staining, and TUNEL staining. TUNEL assays were conducted using the DeadEnd^TM^ Fluorometric TUNEL system (Promega, Madison, WI, USA, G3250) following the manufacturer’s instructions. Images were visualized using a Leica TCS SP8 confocal microscope. The primary antibody was used: PCNA (mouse, 1:50, Santa Cruz, CA, USA).

### 2.7. Real-Time PCR

Briefly, 1 µg of total RNA was transcribed by using Reverse Transcriptase Kit (Promega, A3500) following the manufacturer’s instructions. Real-time PCR was conducted by using the Roche lightcycle 480 Real-time PCR system. Data were calculated using the formula 2^–ΔΔCt^ and normalized with the housekeeping gene GAPDH. Primers used for RT-PCR are shown in the Supplemental Table S1.

### 2.8. Transcriptomic Sequencing

Total RNA was extracted in accordance with the manufacturer’s instructions. The primary procedures for transcriptome sequencing include RNA quantification and qualification, library preparation, clustering and sequencing, and data analysis. HTSeq v0.6.0 was utilized to count the number of reads mapped to each gene. The FPKM of each gene was calculated on the basis of the length of the gene and the read count mapped to the gene. The differential expression analysis of the two groups was performed with the DESeq2 R package (1.10.1). GO enrichment analysis was conducted on differentially expressed genes with the clusterProfiler R package. The GSEA web interface with the Molecular Signatures Database was applied to reveal significantly enriched signaling pathways. Adjusted *p* < 0.05 was considered to indicate significantly differential expression. Liebing Biomedical Technology Co., Ltd. (Shanghai, China) supported the transcriptome sequencing in our research.

### 2.9. 5α-Reductase Activity Inhibition Assay

For the 5α-reductase inhibition assay, female Sprague-Dawley rats (8 weeks old, 250 ± 30 g) were sacrificed. Liver tissues were immediately excised and homogenized in ice-cold buffer (0.32 M sucrose, 1 mM DTT, 1 mM EDTA, 20 mM PBS, pH 5.5) using a glass homogenizer at a 1:4 (*w*/*v*) tissue-to-buffer ratio. The homogenate was centrifuged at 10,000× *g* for 15 min at 4 °C, and the resulting supernatant was further ultracentrifuged at 100,000× *g* for 1 h at 4 °C to obtain the microsomal fraction. The microsomal pellet was resuspended and stored at −80 °C until use. The enzymatic reaction was performed in a 1.5 mL system containing 500 μL PBS (20 μM, pH 6.5), 100 μL test compound (50% ethanol, finasteride, or TCFE), 150 μL testosterone (0.5 mg/mL), 250 μL NADPH (2.0 mg/mL), and 500 μL microsomal preparation. After 30 min incubation at 37 °C in the dark, the reaction was terminated with ice-cold methanol and centrifuged at 1000× *g* for 5 min at 4 °C. The supernatant was filtered (0.22 μm) and analyzed spectrophotometrically at 340 nm to determine NADPH consumption, from which the 5α-reductase II inhibition rate was calculated.

### 2.10. Isolation and Culture of Human Hair Follicles and Scalp Tissues

Occipital scalp specimens were obtained from patients undergoing cosmetic procedures at the Department of Dermatology and Plastic Surgery, Xinhua Hospital (Shanghai, China), following informed consent and institutional ethical approval. All procedures were performed under sterile conditions in a Class II biological safety cabinet. Excised scalp tissues were immediately placed in 6 cm culture dishes containing PBS supplemented with 1% penicillin/streptomycin. Intact hair follicles were identified under a stereomicroscope and carefully dissected using sterile surgical instruments. Isolated hair follicles were photographed for morphological documentation before being transferred to 24-well plates containing 400 μL of complete culture medium (Williams’ E Medium supplemented with 0.02 mM L-glutamine, 0.1 μg/mL insulin, 10 ng/mL hydrocortisone, and 100 μg/mL penicillin/streptomycin). Experimental groups received TCFE at specified concentrations, while control groups received vehicle alone. Cultures were maintained at 37 °C in a humidified 5% CO_2_ incubator for 7 days, with medium replacement on day 4. Scalp tissue explants were processed using identical culture protocols.

### 2.11. Statistical Analysis

Data are expressed as mean ± SEM. An unpaired Student’s *t*-test was used to analyze data sets between two groups. * *p* < 0.05, ** *p* < 0.01 and *** *p* < 0.001 indicated a significant difference. Statistical calculations were performed using GraphPad Prism 5.

## 3. Results

### 3.1. TCFE Enhances the Activity of Wnt Signaling, TGF-β Signaling, and Steroid Hormone Metabolism in DPCs

TCFE was obtained via aqueous extraction, followed by filtration, collection of the filtrate, and drying, yielding the final TCFE product with a 30–50% extraction yield. The extract was characterized by HPLC-MS, and the resulting total ion chromatogram (TIC) revealed that phenols and terpenes accounted for 65–75% of the relative content in the TCFE sample (Figure S1).

**Figure 1:**
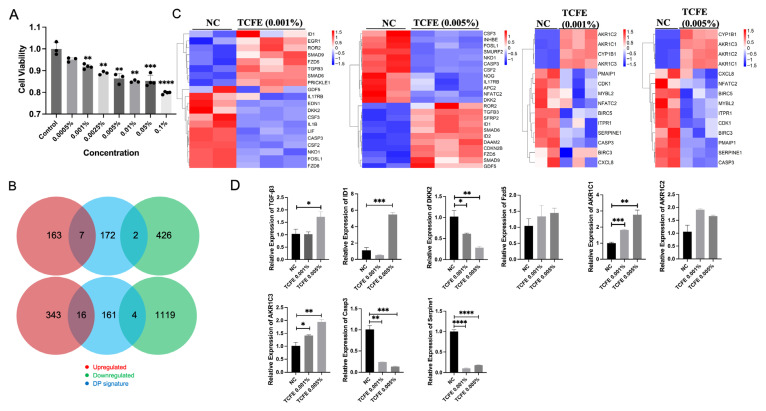
TCFE enhances the activity of Wnt signaling, TGF-β signaling, and steroid hormone metabolism in DPCs. (**A**) Effect of different concentrations of TCFE on DPCs viability. Data are presented as the mean ± SEM compared with Control group. (**B**) Changes in Biomarkers in DPCs (From top to bottom, they are Negative Control vs. TCFE 0.001% and Negative Control vs. TCFE 0.005%, respectively). (**C**) Heat maps of DEGs in the TGF-β signaling pathway (left) and steroid hormone metabolism (right). Red indicates upregulation and blue indicates downregulation. NC, negative control. (**D**) RT-PCR validation of some DEGs. Data are presented as the mean ± SEM, * *p* < 0.05, ** *p* < 0.01, *** *p* < 0.001, **** *p* < 0.0001.

In order to investigate the effect of TCFE on DPCs, we conducted transcriptomic sequencing. First, the proper concentration of TCFE was determined by CCK-8 cytotoxicity testing. The result showed that the effect of TCFE on cell viability was concentration-dependent, where the cell viability decreased as the concentration of TCFE increased (Figure 1A). We then set the TCFE concentration range that ensures a cell viability of 80% or above as the safe range, with the concentration of 0.05% being preliminarily considered as the safety threshold.

Subsequently, high-throughput sequencing was used to investigate the mRNA expression profiles in the Negative Control (NC), TCFE-0.001% (Hezi0001), and TCFE-0.005% (Hezi0005) groups. Each group had three replicates, and a total of nine DPC samples were collected. According to the Pearson correlation heatmap, it is evident that the data of NC-1 exhibits a significant deviation from that of NC-2 and NC-3. Consequently, NC-1 was excluded from further analysis (Figure S2A). Differentially expressed genes (DEGs) were screened according to the conditions of log2FC ≥ 1 or ≤ −1 and FDR ≤ 0.05, and a volcano plot was used to display the difference in expression levels of genes in the two groups of samples (Figure S2B). Compared with the NC group, TCFE-0.001% had 170 up-regulated and 428 down-regulated DEGs, while TCFE-0.005% had 359 up-regulated and 1123 down-regulated DEGs. Notably, a higher concentration of TCFE has a greater impact on the transcriptome of DPCs. Biomarkers of DPCs analysis showed that only few genes related to DPCs were significantly fluctuated, indicating that TCFE has subtle effects on DPCs properties and will not cause abnormal cell differentiation of DPCs (Figure 1B).

The KEGG analysis showed that the TGF-β signaling pathway, the crucial pathway related to hair growth, was stimulated in two TCFE-treated groups (Figure S3A). Heat map of DEGs revealed that the expression of genes involved in the TGF-β signaling pathway, such as *TGF-β3*, *ID1/ID2*, *SMAD6*, and *SMAD9*, was significantly upregulated in the two TCFE-treated groups (Figure 1C). The increased expression of *TGF-β3* and *ID1* was further validated by RT-PCR (Figure 1D). *ID1* and *ID2* are downstream target genes of *SMAD1/5/8* that are highly transcribed in hair follicle stem cells and involved in hair follicle stem cell fate determination [28]. *SMAD6* functions as a negative regulator of the BMP signaling. It inhibits BMP signal activation through competitively binding to the type I BMP receptor, or competing with SMAD4 for interaction with activated SMAD1/5 [29]. Inhibition of BMP signaling in dermis shortens the telogen phase and accelerates the entry into the anagen phase [30]. *SMAD9* is known as *SMAD8*, like *SMAD1/5,* which usually performs as a transcription factor for TGF-β signaling. In Treg cells, glucocorticoid receptor and FOXP3 synergistically induce *TGF-β3* and activate SMAD2/3 to promote the proliferation of hair follicle stem cells [31]. Overall, the TGF-β signaling pathway was upregulated in response to TCFE, suggesting that TCFE may increase the hair inductivity of DPCs through activating the TGF-β signaling pathway. Meanwhile, we found that *DKK2*, a Wnt signaling inhibitor, was down-regulated in both TCFE groups (Figure 1C,D). The expression profile of *DKK2* can affect the density of guard hair [32]. In the group of TCFE-0.005% vs. NC, except for the downregulation of *DKK2*, the receptor *FZD5* of Wnt signaling was upregulated (Figure 1C,D), fully indicating that the Wnt signaling pathway was activated under TCFE treatment.

Interestingly, KEGG analysis showed that steroid hormone metabolic process and steroid hormone biosynthesis pathway were upregulated in both TCFE groups (Figure S3A,B), and correspondingly, the expressions of *AKR1C1/2/3* were significantly upregulated (Figure 1C,D). Aldo-keto reductase (AKRs) is a member of the oxidoreductase superfamily. The AKRs encoded by *AKR1C1/2/3* have the ability to metabolize testosterone and DHT [33]. Activation of the genes in the *ARK1C* family related to androgen metabolism in the TCFE-treated DPCs, indicating a potential capacity of TCFE to promote the metabolism of testosterone or DHT. Among inflammation-related targets, we identified the repressed IL-17 and TNF signaling pathways in TCFE-treated groups based on KEGG analysis (Figure S3A). Heat map analysis showed that TCFE strongly inhibited the expression of the inflammatory signaling molecules, such as *IL17RE*, as well as the cell senescence and apoptosis-related genes, such as *SERPINE1* and *Caspase3* (*CASP3*) (Figure 1C), which was further supported by RT-PCR (Figure 1D), indicating the protective effect of TCFE on DPCs.

In summary, TCFE showed some positive effects in DPCs on multiple levels, such as upregulating the Wnt and TGF-β signaling, attenuating cell apoptosis and senescence, and reducing the stress of steroid hormones by upregulating *AKR1C* family expression.

### 3.2. TCFE Protects Cultured DPCs from Testosterone-Induced Damage via 5α-Reductase Inhibition and AKR1C Gene Upregulation

**Figure 2:**
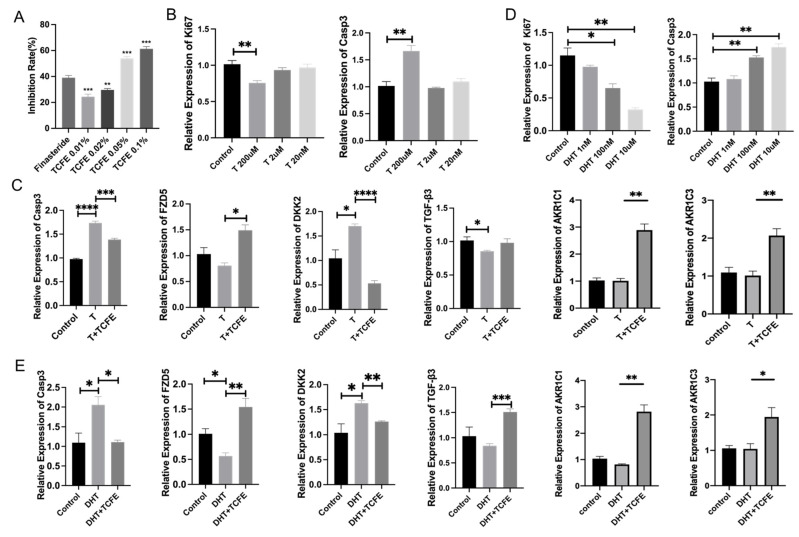
TCFE attenuates testosterone-mediated suppression of DPCs viability through 5α-reductase inhibition and steroid hormone metabolism. (**A**) Inhibitory effect of TCFE on type II 5α-reductase, compared with finasteride. (**B**) Effects of different T concentrations on Ki67, Casp3 expression in DPCs. (**C**) Expression of related genes in DPCs treated by TCFE (0.005%) and T (200 μM), statistical comparisons were performed between the indicated groups (Control vs. T, and T vs. T+TCFE). (**D**) Effects of different DHT concentrations on Ki67, Casp3 expression in DPCs. (**E**) Expression of related genes in DPCs treated by TCFE (0.005%) and DHT (100 nM); statistical comparisons were performed between the indicated groups (Control vs. DHT, and DHT vs. DHT+TCFE). Data are presented as the mean ± SEM, * *p* <0.05, ** *p* <0.01, *** *p* < 0.001, **** *p* < 0.0001.

Testosterone is converted into DHT through the catalysis of 5α-reductase II. Therefore, inhibiting 5α-reductase activity represents a potential therapeutic strategy for AGA. To investigate the effect of TCFE on 5α-reductase, we established an in vitro 5α-reductase enzymatic reaction system. TCFE at 0.05% and 0.1% concentrations exhibited a strong ability to inhibit 5α-reductase of nearly 60%, which was even higher than finasteride (Figure 2A). We subsequently checked the effect of TCFE on DPCs with Testosterone addition. First, the sensitivity of DPCs to the Testosterone hormone was tested, and the evaluation indicators included the proliferation molecule Ki67 and the apoptosis molecule Caspase3. The result showed that 200 μM of Testosterone caused significant differences compared with the control (Figure 2B). The concentration of TCFE was set as 0.005% based on the RNA-seq results. As shown in Figure 2C, the addition of Testosterone significantly activated the expression of apoptotic gene *Caspase3*, which could be reversed by TCFE. The upregulation of Wnt inhibitor gene *DKK2* and downregulation of Wnt receptor gene *FZD5* both suggested that Wnt signaling was inhibited by Testosterone, and this inhibition was significantly relieved by the addition of TCFE. Similarly, the expression of *TGF-β3* was upregulated with the addition of TCFE. These results implied that TCFE probably inhibited the conversion of Testosterone to DHT by inhibiting the activity of 5α-reductase, and finally alleviated the effect of Testosterone on DPCs. We also assessed the effect of TCFE on DPCs treated directly with 100 nM DHT (Figure 2D). Obviously, TCFE could also improve the negative effects of DHT on DPCs, including inhibition of apoptosis, activation of Wnt signaling, and TGF-β signaling (Figure 2E). Interestingly, we similarly observed significant upregulation of *AKR1C1* and *AKR1C3* gene expression in both experiments (Figure 2C,E), consistent with the previous RNA-seq results. These results demonstrate that TCFE not only reduces testosterone conversion to DHT (thereby protecting DPCs) through 5α-reductase inhibition, but also enhances DHT metabolism Via *AKR1C* gene upregulation, collectively mitigating DHT-induced damage to DPCs.

### 3.3. TCFE Can Reverse the Hair Loss of the AGA Mouse Model

**Figure 3:**
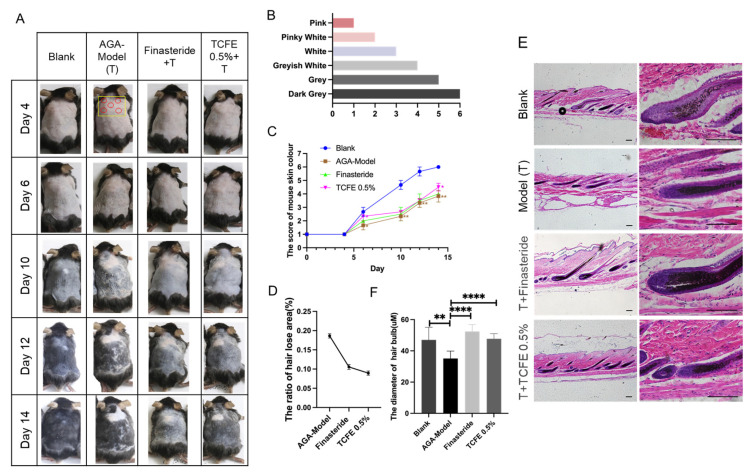
TCFE reverses the hair loss of the AGA mouse model. (**A**) Effects of TCFE on AGA mice (*n* = 3 each group, red circle indicates injection sites, yellow square denotes quantified regions). (**B**) Mouse skin color score. (**C**) Mouse skin color score curve. (**D**,**E**) The proportion of hair lost area in mice (hair lost area/hair shaving area) and the diameter of hair bulb (*n* ≥ 20) in the section (Scale bar = 50 μm). (**F**) HE staining images of hair follicle sections from the alopecic area in AGA mice. Statistical comparisons were performed between the indicated groups (Blank vs. AGA-Model, and AGA-Model vs. Finasteride/TCFE 0.5%). Data are presented as the mean ± SEM, * *p* <0.05, ** *p* < 0.01, *** *p* < 0.001, **** *p* < 0.0001.

To further evaluate the in vivo hair regeneration activity of TCFE, telogen dorsal skins of C57BL/6 mice were treated with testosterone propionate for 14 days to construct an AGA mouse model. Throughout this period, AGA mice were administered finasteride orally or TCFE topically (Figure 3A). According to Fu’s study [34], we used the degree of skin pigmentation as evidence for assessing hair growth stages, ranging from bright pink in the telogen phase to gray/black in the anagen phase (Figure 3B). Under the influence of testosterone propionate, mice exhibited a global or local hair growth retardation on the back. The skin color score of the AGA group was significantly lower than that of the blank group, while the scores of the finasteride group and the TCFE group were both higher than the AGA group (Figure 3C). It is worth noting that compared to finasteride, TCFE can quickly promote hair into the anagen phase on the 5th to 6th day. In addition to color scoring, the severity of AGA was evaluated by setting the grayscale threshold of the image and the ratio of the mouse hair loss area to the total area. After treatment with finasteride or TCFE, the proportion of hair loss areas (hair lost area/hair shaving area) was significantly reduced (Figure 3D). In addition, skin sections were taken for HE staining to detect the status of hair follicles. It was found that hair follicles in the AGA group mainly stayed in the catagen stage, while follicles in other groups had entered in anagen stage (Figure 3E). Obviously, the average diameter of hair bulb in the finasteride group and TCFE group was significantly higher than that in the AGA group (Figure 3E,F).

**Figure 4:**
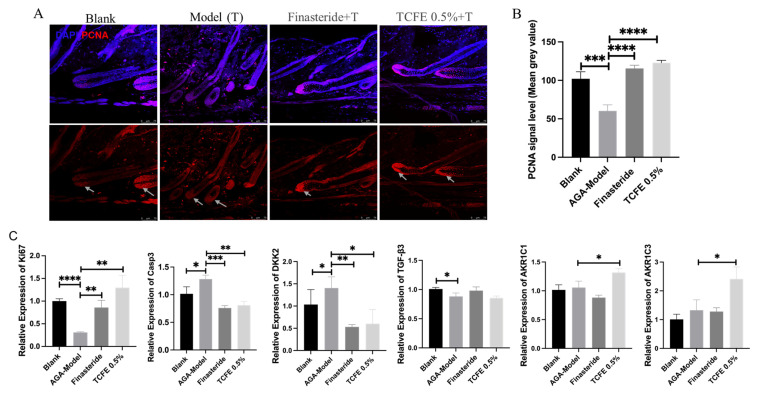
TCFE stimulates hair regrowth in AGA mice through multi-pathway activation. (**A**) Immunofluorescence staining of hair follicle sections from the alopecic skin area in AGA mice, demonstrating DAPI (blue) and PCNA (red). The white arrowhead marks the site of PCNA enrichment (Scale bar = 75 μm). (**B**) Quantitative analysis of PCNA signal intensity (mean gray value). (**C**) Effects of TCFE on the expression of *Ki67*, *Casp3*, *DKK2*, *TGF-β3*, *AKR1C1*, and *AKR1C3* in dermal cells of AGA mice. In B and C, statistical comparisons were performed between the indicated groups (Blank vs. AGA-Model, and AGA-Model vs. Finasteride/TCFE 0.5%). Data are presented as the mean ± SEM, * *p* < 0.05, ** *p* < 0.01, *** *p* < 0.001, **** *p* < 0.0001.

PCNA immunofluorescence staining showed that cell proliferation was reduced upon testosterone propionate administration, but effectively recovered in the finasteride group and the TCFE group (Figure 4A,B). Real-time PCR analysis for dermal tissues further showed that the expression of *Ki67* was significantly downregulated, while *Caspase3* was significantly upregulated in the AGA model (Figure 4C). After treatment with finasteride or TCFE, the proliferation and apoptosis of dermal cells were restored to a normal extent. Although *TGF-β3* expression was not improved evidently in the two administration groups, *DKK2*, the Wnt inhibitory molecule, was significantly increased in the AGA model and reduced in the finasteride and TCFE groups (Figure 4C), indicating that the dysfunction of Wnt signaling could be relieved in dermal cells by finasteride and TCFE. Interestingly, *AKR1C1* and *AKR1C3* were specifically upregulated in the TCFE treatment group, but not in the finasteride group (Figure 4C), suggesting a unique mechanism of TCFE in modulating these aldo-keto reductases. From an animal model perspective, it is suggested that TCFE may not only activate Wnt signaling in dermal cells but also consume intracellular DHT by activating the metabolic reaction of androgens, which could potentially alleviate the pressure of DHT on hair follicles, ultimately relieving AGA.

### 3.4. TCFE Can Reverse DHT Repression on the Growth of Human Hair Follicle

**Figure 5:**
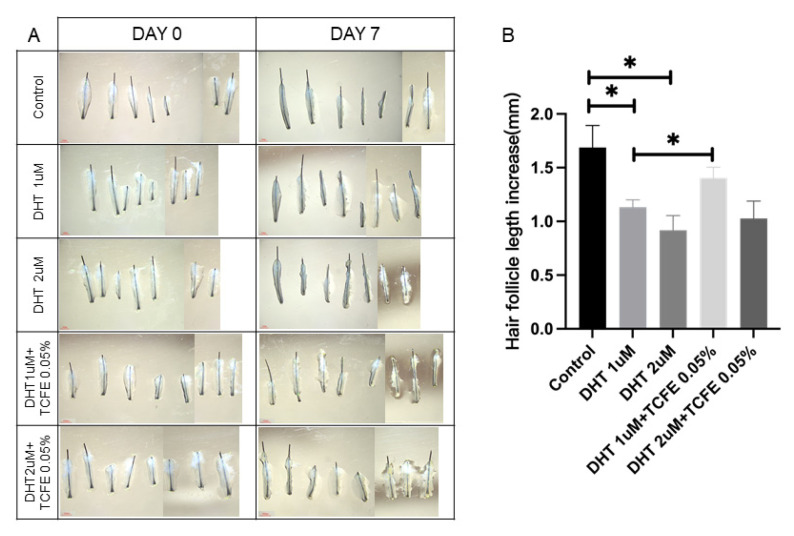
TCFE counteracts DHT-induced growth inhibition in human hair follicles. (**A**) Cultured human hair follicles in vitro (Scale bar = 1 mm). (**B**) Quantification of hair shaft elongation after 7 days of culture. Statistical comparisons were performed between the indicated groups (Control vs. DHT 1 μM/DHT 2 μM, DHT 1 μM vs. DHT 1 μM + TCFE 0.05%, and DHT 2 μM vs. DHT 2 μM + TCFE 0.05%). * *p* < 0.05.

To investigate the effect of TCFE on the growth of human hair follicles, intact hair follicles were isolated from the healthy scalp and continuously cultured for 7 days. After treatment with DHT, hair growth in two concentrations (1 μM and 2 μM) was significantly repressed, which was consistent with the inhibitory effect of androgens on hair growth (Figure 5A,B). After adding 0.05% TCFE, hair growth in both DHT groups was restored, especially the effect of the group with 1 μM DHT + TCFE was remarkable.

**Figure 6:**
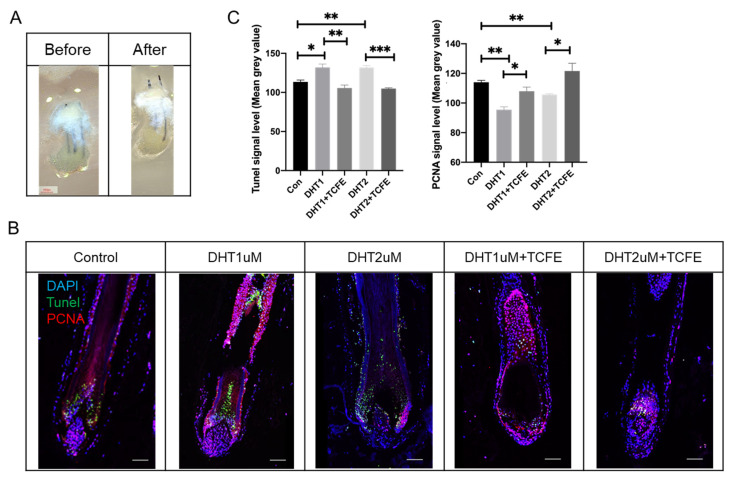
TCFE promotes the growth of human hair follicles in scalp tissue. (**A**) Cultured human scalp tissue with hair follicles (Scale bar = 1 mm). (**B**) Cultured human scalp tissue with hair follicles in DHT (1 μM or 2 μM) and TCFE (0.05%) condition (Scale bar = 75 μm). (**C**) Quantitative analysis of TUNEL and PCNA signal intensity (mean gray value). Statistical comparisons were performed between the indicated groups (Con vs. DHT 1 μM /DHT 2 μM, DHT 1 μM vs. DHT 1 μM + TCFE, and DHT 2 μM vs. DHT 2 μM + TCFE). Data are presented as the mean ± SEM, * *p* < 0.05, ** *p* < 0.01, *** *p* < 0.001.

Furthermore, the human scalp tissues with hair follicles were dissected and cultured in the same way as the isolated hair follicles for 7 days (Figure 6A), then sectioned for immunofluorescence staining for PCNA and TUNEL staining. It can be observed that PCNA was mainly expressed in the dermal papilla (DP) and matrix cells in the hair bulb, and also partially expressed in the root sheath (Figure 6B), while the TUNEL signal was enriched in the matrix cells in the hair bulb and hair shaft. Obviously, the apoptosis was increased and the proliferation was reduced after DHT addition. After TCFE treatment, both apoptosis and proliferation were reversed significantly (Figure 6B,C). In summary, TCFE plays a positive role in promoting hair growth in human hair follicle culture.

## 4. Discussion

To address the limitations of current FDA-approved AGA therapeutics (e.g., minoxidil and finasteride), which are often associated with undesirable side effects, natural product-based herbal therapies have emerged as promising alternatives. Several botanicals, including saw palmetto (*Serenoa repens*), green tea (*Camellia sinensis*) polyphenols, pumpkin (*Cucurbita pepo*) seed oil, rosemary (*Rosmarinus officinalis*) oil, and licorice (*Glycyrrhiza glabra*) root extract, have demonstrated clinical efficacy as 5α-reductase inhibitors [16]. Furthermore, traditional medicinal plants such as *Polygonum multiflorum* (He shou wu) and *Rumex japonicus* have been shown to promote anagen phase progression through Wnt/β-catenin pathway activation [35,36]. Our research reveals that *Terminalia chebula* fruit extract (TCFE) exerts potent hair growth-stimulating effects across both in vivo models and ex vivo human tissue systems. Mechanistically, TCFE appears to combat AGA through tripartite pathway regulation: (1) activation of Wnt and TGF-β signaling cascades, (2) enhancement of androgen metabolic clearance via AKR1C enzyme family upregulation, and (3) direct inhibition of 5α-reductase activity.

Androgens, particularly DHT, play a pivotal role in AGA development through their interaction with AR in hair follicles [4]. DHT is converted from testosterone Via 5α-reductase. DHT then binds to androgen receptors on DPCs, suppressing DPCs’ activity and inducing the expression of autocrine and paracrine inhibitory cytokines, including *TGF-β1*, *DKK1*, and *IL-6* [37,38,39]. This process subsequently inhibits the proliferation of hair follicle epithelial cells, promotes their apoptosis, and ultimately leads to hair loss. Currently, 5α-reductase inhibitors that suppress the conversion of testosterone into DHT are widely used to treat AGA [40,41], essentially reducing DHT levels. Our study reveals that TCFE significantly upregulates *AKR1C* family gene expression, particularly *AKR1C1* and *AKR1C3*, in both cell-based assays and murine skin tissues. The AKR1C protein family, functioning as isoforms of 3α-hydroxysteroid dehydrogenase (3α-HSD), can bind to DHT [42] and degrade DHT by acting as a 3-ketosteroid reductase in the presence of NADPH [43]. These findings suggest TCFE may mitigate DHT-induced damage to DPCs and hair follicles by enhancing AKR1C-mediated DHT metabolism. Prior research has confirmed the AKR1C gene family as key therapeutic targets for AGA. Sulforaphane (SFN), an isothiocyanate derived from broccoli, has been identified as a potential regulator that promotes DHT degradation. Studies have shown that SFN upregulates DHT-metabolizing enzymes, including *AKR1C1* [44] and *AKR1C2* [45], in human cultured cells. Moreover, SFN enhances systemic DHT clearance, thereby mitigating DHT-induced hair growth inhibition [46]. In the present study, TCFE exhibits a comparable mechanism by upregulating *AKR1C* family gene expression and demonstrates potential to reverse AGA progression. These findings suggest that the *AKR1C* gene family may serve as critical therapeutic targets for AGA, warranting further investigation.

Among hair growth-related pathways, the Wnt/β-catenin and BMP signaling pathways represent two of the most critical regulatory pathways governing the transition between hair follicle quiescence (telogen) and growth phase (anagen) [47,48]. During telogen, elevated BMP signaling actively maintains HFSC quiescence [30]. As follicles transition from telogen to anagen, BMP signaling undergoes downregulation while Wnt signaling becomes activated [47,49,50]. Activation of Wnt signaling in hair follicle stem cells (HFSCs) promotes β-catenin accumulation and nuclear translocation, where it forms complexes with *TCF/Lef* transcription factors to activate the expression of genes governing cell proliferation, differentiation, and hair cycle regulation [50,51,52]. Genetic studies demonstrate that β-catenin overexpression induces HFSC proliferation and precocious anagen entry, whereas β-catenin knockout blocks HFSC proliferation and inhibits anagen initiation [53]. Notably, androgens exert inhibitory effects on Wnt/β-catenin signaling, thereby contributing to AGA progression [54]. The TGF-β signaling pathway, particularly through its Smad2/3-dependent mechanism, represents another critical modulator of hair follicle biology. Injection of TGF-β1 into the back skin of mice induced premature catagen development [55]. Conversely, embryonic skin explants with TGF-β2 protein beads induced hair follicle development [56]. Actually, during the telogen-to-anagen transition, DPCs express and secrete TGF-β2 protein, which reaches HFSCs and transiently activates Smad2/3 signaling in HFSCs, coinciding with the initiation of tissue regeneration [57], which implies that different TGF-β isoforms play different roles during hair cycling. In our current study, TCFE consistently activated Wnt signaling (evidenced by increased *FZD5* and decreased *DKK2* expression) in both cellular assays and AGA animal models. Notably, we observed upregulated TGF-β3 expression in TCFE-treated DPCs, suggesting that DP-derived TGF-β3 may share hair growth-promoting properties with TGF-β2. We also noted that no significant upregulation of *TGF-β3* was observed in the animal models. We speculate that this may be attributed to the use of bulk dermal tissues rather than purified DPCs in our experiments, where cell-type-specific expression changes could have been masked by signals from the overall tissue. Although prior studies reported no discernible impact of TGF-β3 knockout on hair development or cycling [56], we propose that functional redundancy among TGF-β isoforms may compensate for its loss.

Very recently, Hong et al. [58] reported that *T. bellirica* extracts reversed AGA by activating the MAPK signaling pathway and remodeling the follicular microenvironment through antioxidative and pro-angiogenic effects. Although *Terminalia chebula* and *Terminalia bellirica* are phylogenetically related plants from the Combretaceae family and *Terminalia* genus—sharing similar chemical constituents and the ability to inhibit type II 5α-reductase, their mechanisms of promoting hair growth differ significantly. In our work, we demonstrate that TCFE promotes hair growth via distinct pathways: it activates Wnt/β-catenin and TGF-β signaling, and uniquely dual modulates DHT by both inhibiting its synthesis from testosterone and enhancing its metabolic clearance Via *AKR1C* gene upregulation. This combined action mitigates DHT-induced damage and counteracts early-stage androgenetic alopecia pathophysiology, revealing a mechanism fundamentally different from that of *T. bellirica*.

## 5. Conclusions

In summary, TCFE promotes the proliferation of hair follicle epithelial cells and stimulates hair growth by activating both the Wnt signaling pathway and TGF-β3 signaling in DPCs. Concurrently, TCFE inhibits the onset of AGA through suppressing type II 5α-reductase activity. Most notably, our findings suggest that TCFE may accelerate DHT metabolism by upregulating *AKR1C* gene expression, thereby mitigating DHT-induced damage to DPCs and consequently alleviating androgen-mediated hair loss phenotypes. However, several critical questions remain to be addressed in future studies: First, we need to identify which specific monomeric component(s) within TCFE are responsible for its hair growth-promoting and AGA-resistant effects. Second, the precise molecular mechanisms through which these active compounds exert their functions require further elucidation.

## Data Availability

All data generated or analyzed during this study are included in this article. Further enquiries can be directed to the corresponding author.

## Supplementary Materials

The following supporting information can be downloaded at: https://www.mdpi.com/article/10.3390/biomedicines13112584/s1, Figure S1: HPLC-MS analysis on TCFE; Figure S2: RNA-sequencing analysis-1 for DPCs treated with TCFE; Figure S3: RNA-sequencing analysis-2 for DPCs treated with TCFE; Table S1: primer list used for RT-PCR.

## Abbreviations

The following abbreviations are used in this manuscript:

AGA	Androgenetic alopecia
DHT	Dihydrotestosterone
DP	Dermal papilla
DPCs	Dermal papilla cells
TCFE	*Terminalia chebula* fruit extract
AR	Androgen receptor
H&E	Hematoxylin and eosin
HFSCs	Hair follicle stem cells
