# Supplementary material for: Terminalia chebula Retz. Fruit Extract Promotes Murine Hair Growth by Suppressing 5α-Reductase and Accelerating the Degradation of Dihydrotestosterone
Source: Biomedicines. 2025 Oct 22;13(11):2584. doi: 10.3390/biomedicines13112584 (PMC12650287; doi:10.3390/biomedicines13112584)
Supplement: Supplementary file 1 [file biomedicines-13-02584-s001.zip › biomedicines-3890411-supplementary.pdf]

Figure S1. HPLC-MS analysis on TCFE. TCFE mass spectrometric data acquisition was performed in both positive (**A**) and negative (**B**) ion scanning modes. The resulting total ion chromatogram (TIC) is shown here, where the red, blue, and green traces represent three technical replicates.

Figure S2. RNA-sequencing analysis-1 for DPCs treated with TCFE. A. Pearson correlation heat map. R^2 values from Pearson's correlation analysis were plotted inside the grids of the heatmap. **B.** Volcano plot used to determine the DEGs in Negative Control vs TCFE 0.001% and Negative Control vs TCFE 0.005% with the criteria: log2FC ≥ 1 or ≤ -1 and FDR ≤ 0.05.

Figure S3. RNA-sequencing analysis-2 for DPCs treated with TCFE. A. GO enrichment analysis was conducted on the filtered upregulated and downregulated DEGs respectively. These figures illustrate the top 15 significantly enriched terms at the Biological Process (BP) level of GO annotation. **B.** KEGG Pathway analysis was conducted on the filtered upregulated and downregulated DEGs respectively. These figures illustrate the top 15 most significantly enriched signaling pathways.

Supplemental Table S1 primer list used for RT-PCR

Gene	Species	Primer 5'-3'	
GAPDH	Homo	F	5'-CTGGGCTACACTGAGCACC-3'
		R	5'-AAGTGGTCGTTGAGGGCAATG-3'
ALPL	Homo	F	5'-ACTGGTACTCAGACAACGAGAT-3'
		R	5'-ACGTCAATGTCCCTGATGTTATG-3'
FZD5	Homo	F	5'-CCGTTCGTGTGCAAGTGTC-3'
		R	5'-GAAGCGTTCCATGTCGATGAG-3'
DKK2	Homo	F	5'-TGTACCAAGGACTGGCATTCG-3'
		R	5'-CTGTGGCAATACCTCCCAACT-3'
TGF-β3	Homo	F	5'-GGAAAACACCGAGTCGGAATAC-3'
		R	5'-GCGGAAAACCTTGGAGGTAAT-3'
AKR1C1	Homo	F	5'-CCTAAAAGTAAAGCTTTAGAGGCCACC-3'
		R	5'-GAAAATGAATAAGGTAGAGGTCAACATAAT-3'

AKR1C3	Homo	F	5’-GAGAAGTAAAGCTTTGGAGGTCACA-3’
		R	5’-CAACCTGCTCCTCATTATTGTATAAATGA-3’
Casp3	Homo	F	5’-GAAATTGTGGAATTGATGCGTGA-3’
		R	5’-CTACAACGATCCCCTCTGAAAAA-3’
Serpine 1	Homo	F	5’-GCACCACAGACGCGATCTT-3’
		R	5’-ACCTCTGAAAAGTCCACTTGC-3’
GAPDH	Mus	F	5’-AGGTCGGTGTGAACGGATTTG-3’
		R	5’-TGTAGACCATGTAGTTGAGGTCA-3’
AR	Mus	F	5’-CTGGGAAGGGTCTACCCAC-3’
		R	5’-GGTGCTATGTTAGCGGCCTC-3’
Ki67	Mus	F	5’-AGCACAAAGAGACGGTCTAAGA-3’
		R	5’-CTCTGCCTCGTGACTGTGTT-3’
Casp3	Mus	F	5’-ATGGAGAACAACAAAACCTCAGT-3’
		R	5’-TTGCTCCCATGTATGGTCTTTAC-3’
DKK2	Mus	F	5’-CTGATGCGGGTCAAGGATTCA-3’
		R	5’-CTCCCCTCCTAGAGAGGACTT-3’
TGF-β3	Mus	F	5’-CAGGCCAGGGTAGTCAGAG-3’
		R	5’-ATTTCCAGCCTAGATCCTGCC-3’
AKR1C1	Mus	F	5’-TGCTCTTATAGCCTGTGAGG-3’
		R	5’-AAGGATGACATTCCACCTGG-3’
AKR1C3	Mus	F	5’-TCCAGAGGTTCCAAGAAGTAAAGCTTT-3’
		R	5’-TGGATAATTAGGGTGGCTAGCAAA-3’
