# Supplementary material for: Terminalia chebula Retz. Fruit Extract Promotes Murine Hair Growth by Suppressing 5α-Reductase and Accelerating the Degradation of Dihydrotestosterone
Source: Biomedicines. 2025 Oct 22;13(11):2584. doi: 10.3390/biomedicines13112584 (PMC12650287; doi:10.3390/biomedicines13112584)
Supplement: Supplementary file 1 [file biomedicines-13-02584-s001.zip › supplemental figures.pptx]

## Slide 1
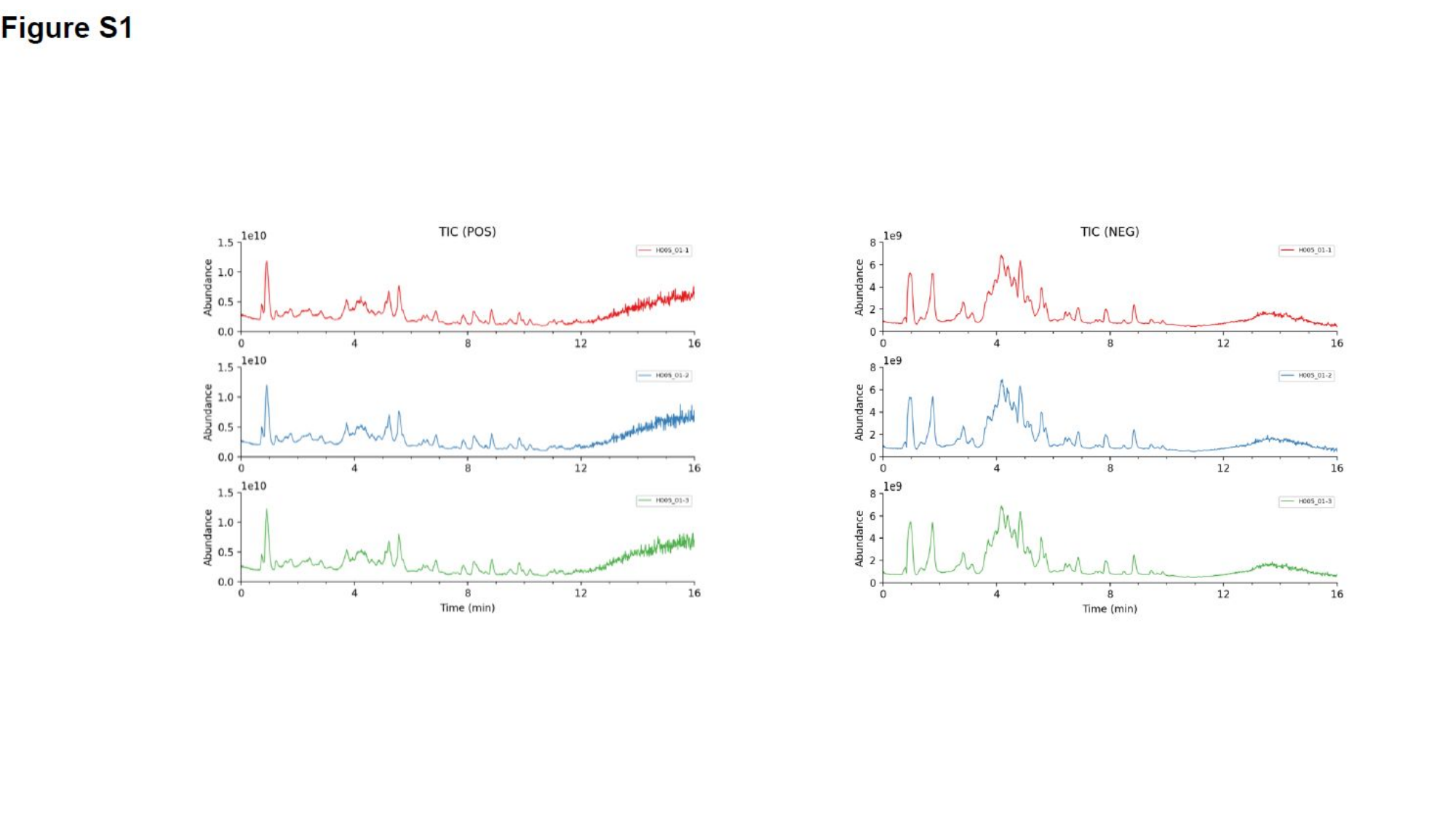

## Slide 2
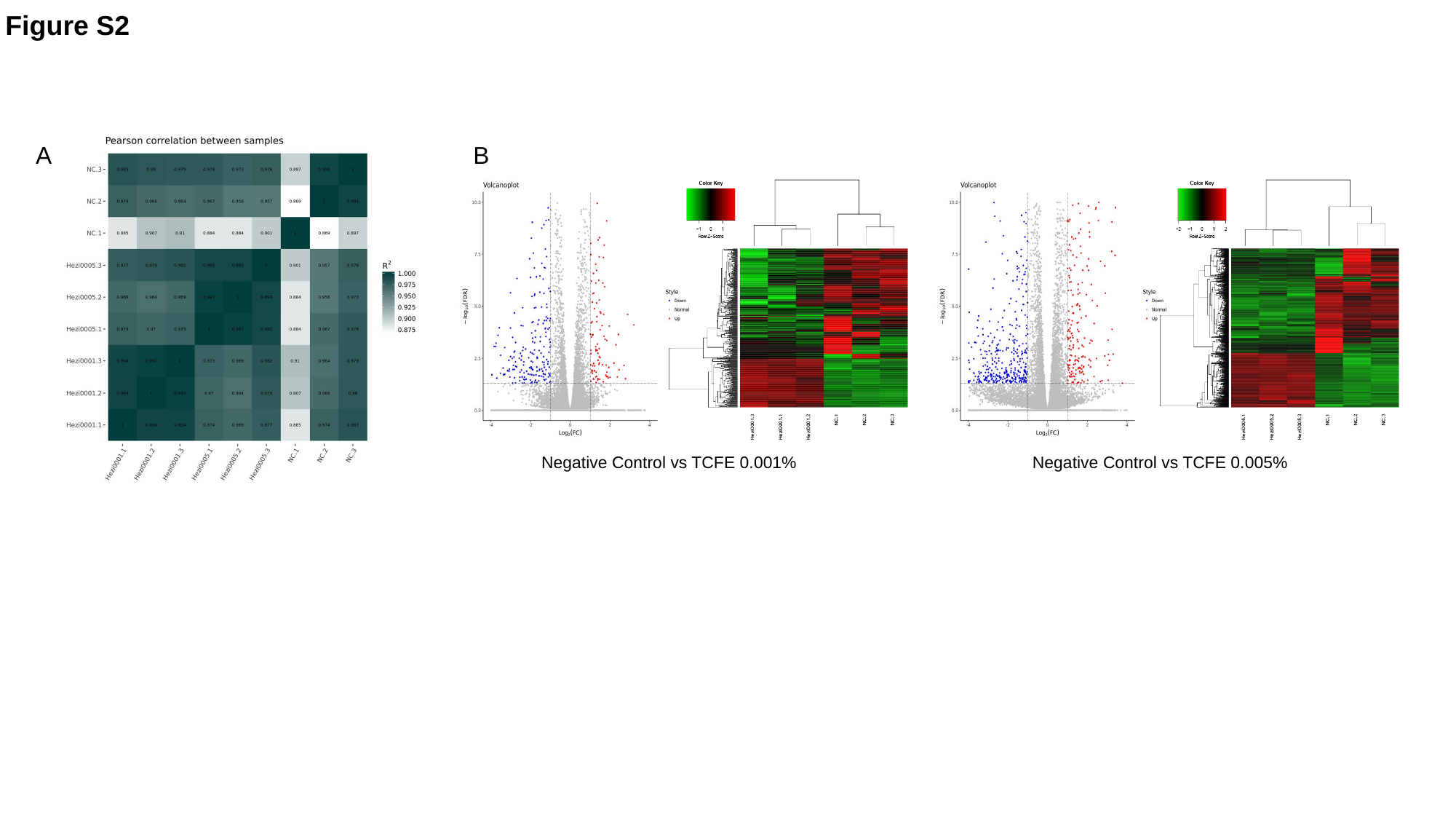

Figure S2
A
B
Negative Control vs TCFE 0.001%
Negative Control vs TCFE 0.005%

## Slide 3
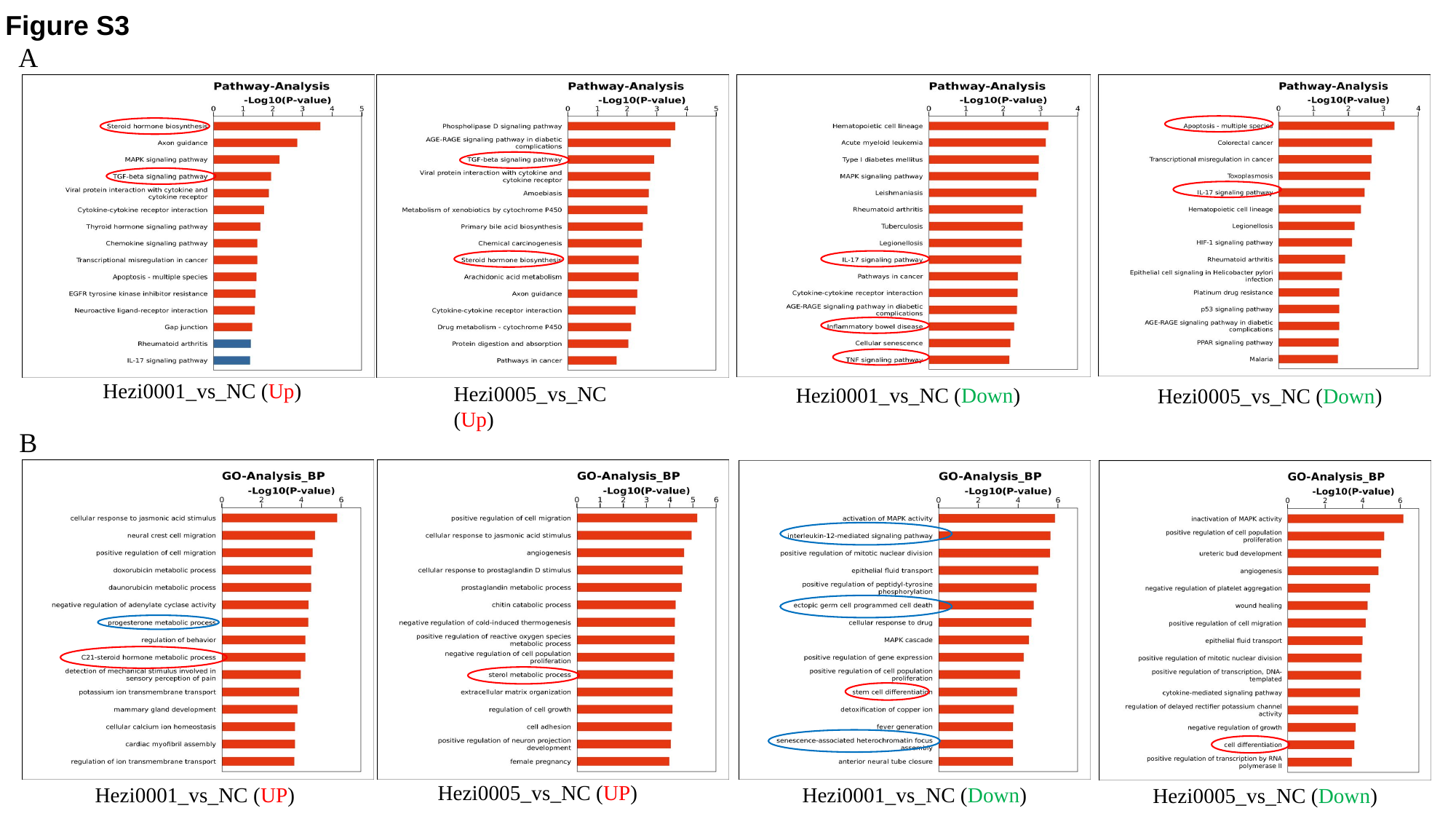

Figure S3
A
Hezi0001_vs_NC (Up)
Hezi0005_vs_NC (Up)
Hezi0001_vs_NC (Down)
Hezi0005_vs_NC (Down)
B
Hezi0005_vs_NC (UP)
Hezi0001_vs_NC (UP)
Hezi0001_vs_NC (Down)
Hezi0005_vs_NC (Down)
